# Alignment Method for Linear-Scale Projection Lithography Based on CCD Image Analysis

**DOI:** 10.3390/s18082442

**Published:** 2018-07-27

**Authors:** Dongxu Ren, Zexiang Zhao, Jianpu Xi, Bin Li, Zhengfeng Li, Huiying Zhao, Lujun Cui, Hang Xu

**Affiliations:** 1 School of Mechatronics Engineering, Zhongyuan University of Technology, Zhengzhou 450007, China; zexiang_zhao@zut.edu.cn (Z.Z.); xjpyq2010@zut.edu.cn (J.X.); libin_zzti@zut.edu.cn (B.L.); zhengfengli@zut.edu.cn (Z.L.); lujun@zut.edu.cn (L.C.); xuhangzzti@zut.edu.cn (H.X.); 2 State Key Laboratory for Manufacturing Systems Engineering, Xi’an Jiaotong University, Xi’an 710049, China; zhaohuiying@mail.xjtu.edu.cn

**Keywords:** alignment method, projection lithography, linear scale, grating linear displacement sensor

## Abstract

This paper presents a method to improve the alignment accuracy of a mask in linear scale projection lithography, in which the adjacent pixel gray square variance method is applied to a charge-coupled device (CCD) image to obtain the best position of the focal length of the motherboard and then realize the alignment of the focal plane. Two image positions in the focal plane of the CCD are compared with the traits overlap according to the image splicing principle, and four typical errors are corrected on the basis of the total grating errors. Simultaneously, the rotation error of the mask is used to summarize the grayscale variation function of the CCD image. Threshold functions are employed to express the factors including the wave crests of the amplitude, period error, and phase error, which govern the rotation accuracy and weight alignment accuracy expression of the established four error factors. Finally, in the experiment, the slope of the mask is corrected and adjusted to the same direction as the slide plate with the assistance of a dual-frequency laser interferometer. The effect of the alignment error on the lithography accuracy is discussed and verified in the static case, and it is found that the CCD maximum resolution pixel is 0.1 μm and accuracy of the scale is 0.79 μm in only a 200-mm-measurement range.

## 1. Introduction

Grating lithography alignment is one of the main influencing methods for improving the accuracy of projection lithography. By the precise focal plane alignment of the projection lithography system, the pattern of a mask is projected accurately on the surface of a scale plane with a positive or negative photoresist, reducing the effect of the alignment error on the accuracy of the grating lithography process in a certain motion range. The alignment of grating lithography is characterized by uniform grating stripes and a certain length; therefore, a major technical problem arises when a lithography plate or mask exhibits a tilting or rotating phenomenon. Such an installation error will seriously affect the accuracy of the linear scale, and some related content has been explained at an international conference [1].

Several lithography methods are used for alignment such as a modified coarse-fine alignment scheme designed for proximity lithography based on a four-quadrant-grating moiré [2,3], $\mathrm{moir}\overset{´}{e}$ fringe precision alignment used in nanoimprint lithography [4], and digital $\mathrm{moir}\overset{´}{e}$ fringe measurement method for alignment in imprint lithography [5]. The optimal high-order alignment adjustment is obtained for maximizing the die yields of semiconductor lithography equipment [6]. However, simply any alignment method cannot be suitable for projection lithography. The alignment marks are selected by analysing the relevant criteria in projection electron lithography [7], and new alignment mark design structures are used for higher diffraction order wafer quality enhancement [8]. All of these lithography methods are not required for large-length range precision alignment.

However, linear scale lithography emphasizes that there should be the uniformity between the lines having the same a 20-μm-pitch. In another study, ultraprecision stages were designed to align the wafers for the microlithography of an integrated circuit [9], which included a wide variety of lines. The different patterns or traits were lithographed on the wafers based on various alignment techniques, but these methods including thermally controlled alignment [10] and wafer rear surface and cancelling tilt effect [11] did not consider the consistency of the lines.

In the large-length range lithography process of the linear scale examined in this study, the alignment error, motion displacement, length of the grating, installation tilt angle of the grating, straightness error of the mobile workbench, and installation precision repeatability have a direct or indirect effect on the alignment accuracy of the linear-scale projection lithography. This in turn affects the lithography accuracy of the linear scale and fewer methods are studied using a charge-coupled device (CCD) based on interferometry. In this study, by performing simulations and experiments, we provide the analysis and verification of the effect of the mask tilt and rotation error on the accuracy of the lithography alignment. The analysis of the image resolution is conducted using CCD images to ultimately achieve a high precision alignment.

## 2. Method

### 2.1. Principle of Accuracy Alignment

**Figure 1:**
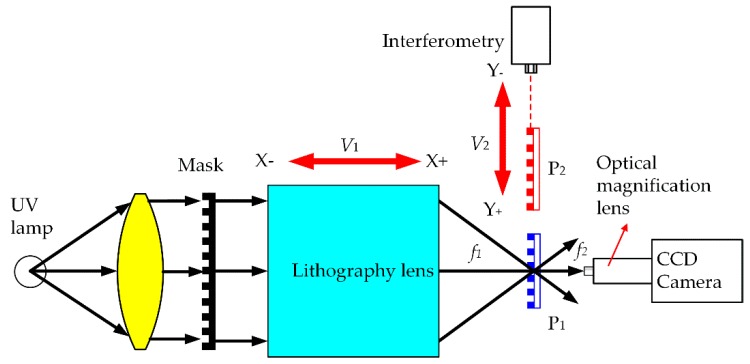
Scheme of the system for the accuracy alignment in projection lithography.

The fundamental principle of projection lithography alignment for high-precision linear scale is illustrated in Figure 1. First, focal length $f_{2}$ of the CCD sensor is adjusted so that it is aligned with the grating focal plane. Second, the custom mask plate is mounted on the projection lithography lens system, the spatial position of the lithography lens is adjusted, and it is ensured that the focal length $f_{1}$ is aligned with the grating focal plane by monitoring the clarity of the CCD image. For the latter, the tilt adjustment method and sharpness algorithm are two important points for achieving alignment. In the next step, the installation mask tilt is adjusted based on the direction of movement of the table, and then the Abbe principle is complied with to align the dual-frequency laser interferometer movement direction and workbench movement in the same direction to ensure that the base surface of the grating and dual-frequency laser interferometer optical path are in a straight line. Simultaneously, the rotation mounting error is adjusted based on a specific algorithm. Finally, precision alignment is achieved by calculating and comparing the gray values of the mask projection image, such as a single pitch and the full width of multiple pitches.

According to some constant distance, a grating graphic window step is performed by another accurate projection lithography to form a linear scale. In Figure 1, *P*_1_ and *P*_2_ represent two different locations achieved by the multi-step movement, and their distance is measured by interferometry. The alignment accuracy can directly affect the precision of the long grating scale. In this study, the grating images were captured by a CCD camera for the alignment accuracy of the depth of focus. The tilt mask was calibrated by interferometry.

**Figure 2:**
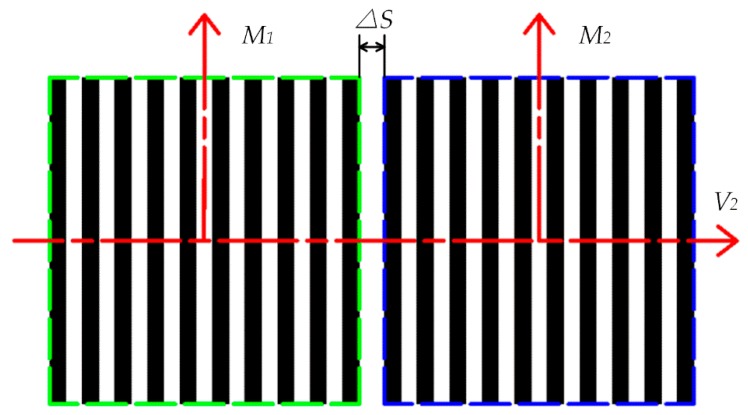
Ideal splicing scheme for two CCD images in the process of precision alignment.

To complete the high-precision grating scale in the lithography manufacturing, the tilt grating on the mask mounted on the lithography lens, a critical part of the alignment accuracy, must be examined, as shown in Figure 2. The gratings located on the front side of the wafers and observed by the CCD optical system exhibit the following position relationship:

(1) $$M_{1}∥M_{2}⊥V_{2}$$

According to Figure 2 and Equation (1), $M_{1}$ and $M_{2}$, which represent the vertical directions of the gratings, are parallel to each other and formed by the two complete separate lithography steps: step I and step II. $V_{2}$ represents the slide body, which is the movement direction of the experimental setup.

### 2.2. Tilt Mask Alignment

In the grating projection lithography process, two key factors have a significant effect on the mask plate alignment, i.e., the precision focus and tilt mask alignment. Moreover, the alignment accuracy and accuracy of the grating are proportional. Owing to the smaller field size of the CCD, one image cannot display the whole grating zone projected by the projection lithography lens. For instance, an actual mask size of 19.2 mm × 19.2 mm will be 4.8 mm × 4.8 mm through the lithography zoom lens. The CCD obtains the image information, and the CCD field of view size 400 μm × 320 μm is transmitted to a computer by an analogue-to-digital signal conversion card. 

**Figure 3:**
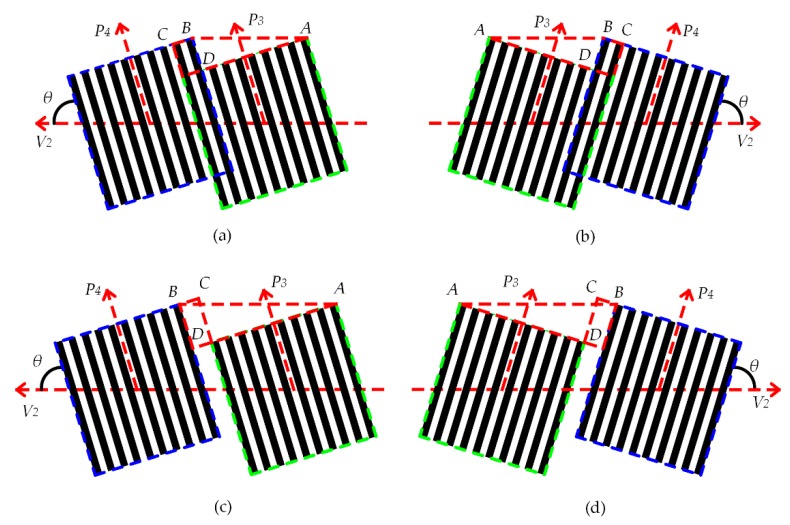
Several grating images obtained at different angles: (**a**) Leaning to the left and overlap phenomenon, overlap value is −*BC/sinθ* (**b**) Leaning to the right and overlap phenomenon, overlap value is −*BC/sinθ* (**c**) Leaning to the left, and the interval distance value is *BC/sinθ* (**d**) Leaning to the right, and the interval distance value is *BC/sinθ*.

Figure 3 shows the several grating images obtained by the different tilt angles in the grating projection lithography process. In the figure, $V_{2}$ represents the direction of the movement of the slide plate on which the photoresist-coated glass plate is mounted. *AB* represents the overlap or interval distance between two adjacent grating projection lithography processes. *θ* represents the angle between grating strips $P_{3}$/$P_{4}$ and $V_{2}$. When *θ* ≠ 90°, the mask projection width is not equal to the distance generated in direction $V_{2}$, which requires the mask alignment error to be eliminated.

Then the tilt angles can be described as

(2) $$\{\begin{array}{c} \theta =arctg\frac{AD}{BD}\\ AB=\sqrt{AD^{2}+BD^{2}} \end{array}$$

Here, the error caused by the tilt angles is

(3) ΔS=BC

The grating is projected *k* times by the lithography lens, and the reduced length is

(4) $$n_{1}\times p+\Delta S$$

where $n_{1}$ is a positive integer, *p* is the pitch of the scale, and $n_{1}\times p$ represents the nominal length of the mask projected on the scale by the lithography lens without considering the errors. The length of the mask is

(5) $$\frac{1}{k}\times \left(n_{1}\times p+\Delta S\right)$$

where *k* represents the lithography lens magnification, which is infinitely close to the ideal value of 0.25. 

The docking error accumulated during the grating projection lithography can be expressed as

(6) $$\sum_{i=0}^{m}\frac{BC}{sin\theta }+\sum_{i=0}^{m}\Delta t_{i}+\sum_{i=0}^{m}\Delta v_{i}+\Delta 3\delta ,\;(m=\frac{L}{n_{1}\times p+\Delta S})$$

where *L* represents the total length of the grating lithography, *m* represents the times of lithography at whole length *L*, $\Delta t_{i}$ represents the *i*-th lithography error caused by the temperature fluctuation, $\Delta v_{i}$ represents the *i*-th lithography error caused by the vibration disturbance, and Δ3δ represents the uncertainty of the measurement feedback.

### 2.3. Rotation Mask Alignment

The rectangular wave with period p is uniformly changed, and the change interval is [0, 255] and amplitude is $A_{m}$, where the rising and falling edges reflect the sharpness. The sharpness is expressed by the amount of the changed angle in the time and space domains; the ideal angle is 90°, which is expressed as the high-frequency components in the frequency domain. Here, it is mainly discussed on the *XY* plane, so that the *X*-direction rectangular wave ideal light intensity can be expressed as

(7) $$I_{1}\left(x,y\right)=A_{m}rect\left(x\right)=\{\begin{array}{c} A_{m},\left(0<x<\frac{p}{2}\right)\\ 0,\left(\frac{p}{2}<x<p\right) \end{array}$$

Due to the numerical aperture, vignetting effect, distortion of the lithography lens, and the existence of focal length alignment error, many high frequency parts are filtered out, that is, the higher harmonic part of the Fourier series is filtered out, and the whole harmonic is filtered. For the loss of higher harmonics, the shape of the rectangular wave projected onto the photoresist plane is not a complete rectangular wave, which is similar to a sine wave, the fundamental signal of the Fourier series of rectangular waves. The simplicity of the fundamental signal makes it easy to reflect the rotation error by simulating the phase and the amplitude.

**Figure 4:**
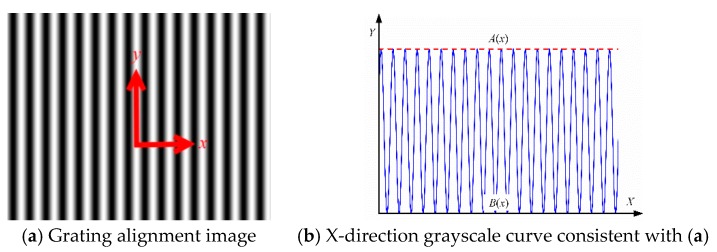
Non-Rotation Mask.

**Figure 5:**
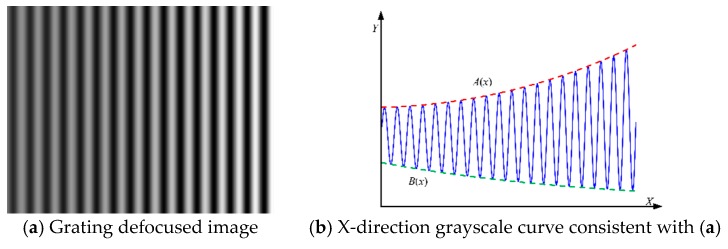
Rotation mask.

Figure 4 and Figure 5 show an example of a non-rotation and rotation mask, respectively. In the actual focusing process, the intensity distribution of the grating photoresist plane cannot be the ideal state because the alignment distance error and *XYZ* axis rotation error will cause amplitude and phase changes. However, if the resolution of the lithography lens system, distortion, and other system errors are not considered, then the light intensity along the *X*-axis direction can be expressed as

(8) $$I_{2}\left(x,y\right)=A\left(x,y\right)I_{1}\left(\omega x+\varphi ,y\right)+B\left(x,y\right)$$

where A(x,y) and B(x,y) can be expressed as the product of a linear function and exponential function and A(x,y) and B(x,y)  represent the change trend of the peak and trough of the gray value curve on the grating image, respectively, which can be expressed as

(9) $$A\left(x,y\right)=\pm k_{1}xexp\left(\alpha_{1}x+\beta_{1}\right)$$

(10) $$B\left(x,y\right)=\pm k_{2}xexp\left(\alpha_{2}x+\beta_{2}\right)$$

where $\pm k_{1}$ represent the trend of the slope slope coefficient, $\pm k_{2}$ indicate the trend of the trough slope coefficient, $\alpha_{1}$ indicates the degree of the increase in the crest index, $\alpha_{2}$ indicates the degree of the increase in the index of the trough, $\beta_{1}$ is the coefficient of the crest exponential function, and $\beta_{2}$ is the coefficient of the valley exponential function.

The correspondence of these parameters is mainly to indicate the rotation of the mask around the *Y*-axis rotation. In the actual grating lithography process, the mask plate grating stripes are rectangular stripes and combined with the above and the expression, the rectangular grating image rotation error can be expressed as

(11) $$I_{2}\left(x,y\right)=\pm A_{m}k_{1}xexp\left(\alpha_{1}x+\beta_{1}\right)rect\left(\omega x+\varphi \right)\pm k_{2}xexp\left(\alpha_{2}x+\beta_{2}\right)$$

where $I_{2}\left(x,y\right)$ is the light intensity function of the grating photoresist plane.

The intensity function of the grating photoresist plane is mainly the variation in the mounting attitude of the mask, such as the verticality, overall rotation angle, gray scale difference on the black and white, and window width error.

As shown in the above Equations (9)–(11), the coefficients such as $\pm k_{1}$, $\pm k_{2}$, $\alpha_{1}$, $\alpha_{2}$, $\beta_{1}$, $\beta_{2}$ are unknown and need to be confirmed, these are the main factors causing the amplitude, phase, and period to become changeable and complex. 

Thus, the complex characterization formula can be simplified, and in the lithography alignment process, the alignment error of the amplitude, phase, and period on functions A(x,y) and B(x,y) will be obtained by the convergence of different alignment accuracies and then completing the different alignment accuracy adjustments. The boundary expressions are as follows:

Amplitude wave errors are

(12) $$\Delta_{1}=MAX\left[A\left(x_{i},y_{i}\right)\right]-A_{m}\approx 0$$

Amplitude trough errors are

(13) $$\Delta_{2}=MAX\left[B\left(x_{i},y_{i}\right)\right]\approx 0$$

Pitch periodic errors are

(14) $$\Delta_{3}=\omega_{i,j}-p\approx 0$$

Phase errors are

(15) $$\Delta_{4}=MAX\left[\varphi_{i,j}\right]-MIN\left[\varphi_{i,j}\right]\approx 0$$

where i and j can be described as $\left\{i\in \left[np,\left(n+1\right)p\right],j\in \left[0,N\right],n\in \left[0,\frac{M}{p}\right]\right\}$.

The phase error reflects the rotation of the image in *XOY*, periodic error reflects the rotation of the mask around the *X* axis, and amplitude error is the most significant factor, where the distance defocus is the main reason for the amplitude change. B(x,y) is more affected by the rotation factor of the mask plate. The value of the above factors directly affects the accuracy of the alignment adjustment.

$\Delta_{1},\;\Delta_{2},\;\Delta_{3}$, and $\Delta_{4}$ are the amplitude wave errors, amplitude trough errors, pitch periodic errors, and phase errors, respectively. These parameters affect the alignment accuracy based on the different weights, which can be expressed as:

(16) $$Alignment\;accuracy=f\left(a\times \Delta_{1},b\times \Delta_{2},c\times \Delta_{3},d\times \Delta_{4}\right)$$

### 2.4. Focal Length Alignment

The CCD image alignment principle, which in essence is that calculation of the grating image collected by the CCD sensor, is based on the utilization of a mathematical evaluation function, reflects the image clarity thought comparing the calculated value, and its clarity is the lithography system focus accuracy.

This section focuses on the clarity of the grating image in the spatial domain; a clear grating image implies a large gray value of the adjacent pixels at the junction of the black and white fringes and more steepness of the graph.

The most commonly used evaluation image clarity functions are the adjacent pixel gray variance method, gray gradient function, statistical function, frequency domain function, and informatics function [12]. Among these, the adjacent pixel gray variance method performance is outstanding in the focus real-time and more effective than the other methods. It can be expressed as

(17) $$F=\frac{1}{n_{2}}\left[\sum \left|G\left(x,y\right)-G\left(x,y-1\right)\right|+\sum \left|G\left(x,y\right)-G\left(x+1,y\right)\right|\right]$$

where $n_{2}$ indicates the total number of pixels of the detected image and G(x,y) corresponds to the gray value of the position point (x,y).

To avoid the effect of the different algorithms and dimension units and better reflect the comparability of the measured data, the pitch data in the CCD graph must be normalized to solve the problem of data comparability. In this study, the min–max standardized normalization method is adopted, which is also known as the standardization of the deviation standardization. Mapping the resulting values to the interval [0, 1] according to the sample library of the collected or calculated data, can be eventually expressed as

(18) $$X^{*}=\frac{x-MIN}{MAX-MIN}$$

where MAX and *MIN* indicates the maximum and minimum value, respectively, in the collected data sample library, *x* represents any pixel value in the interval, and $X^{*}$ indicates the value that has been normalized.

## 3. Experimental Setup

**Figure 6:**
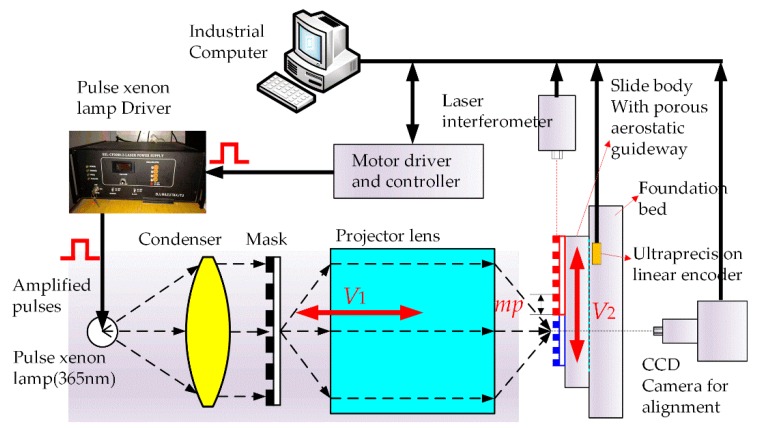
Schematic of the experimental setup.

Figure 6 illustrates the schematic of the experimental setup. The grating lithography alignment system includes a lithography lens, CCD camera for alignment, foundation bed with ultraprecision, side body with porous aerostatic guideway, laser interferometer (Renishaw XL80), and pulse xenon lamp driver, and industrial computer system.

The whole assembly is placed on a precision-foundation dedicated isolation, and the device works in a temperature-controlled room. The lithography machine body is made of a natural granite bed. In Figure 6, $V_{2}$ is the direction of motion which installs porous aerostatic guide rails to ensure a smooth and precise movement. The industrial CCD camera and laser interferometer are mainly used to align before the multi-repeated projection lithography process.

**Figure 7:**
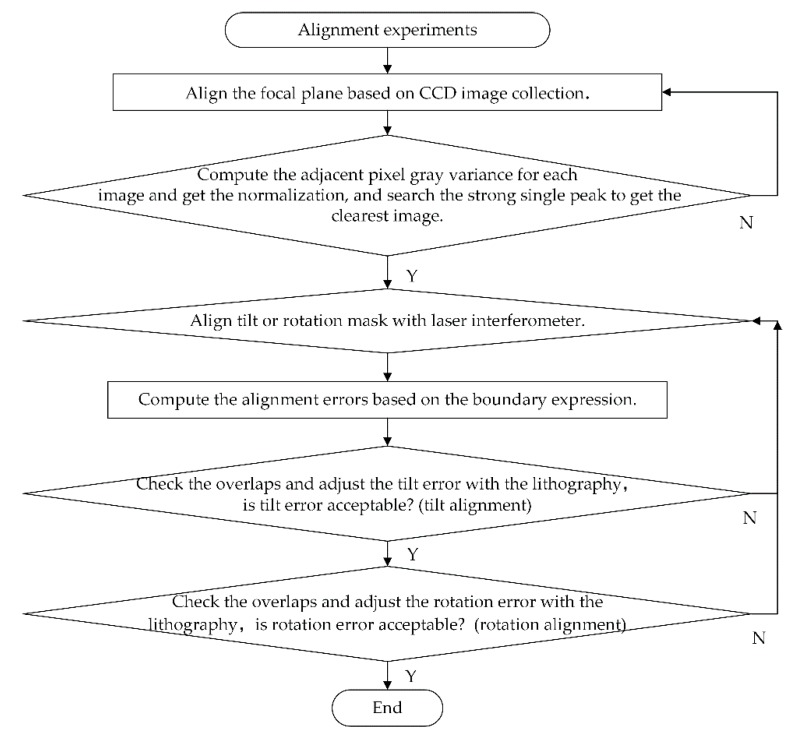
Flowchart of alignment experiments.

Figure 7 shows the flowchart of the alignment experiments, where the tilt error and rotation error are eliminated by the algorithm and lithography experiment platform. First, the focal plane is aligned by collecting the CCD images and searching the strong single peak to obtain the clearest image. For such an image, the adjacent pixel gray variance is computed and normalization is realized. Moreover, the mechanism motion precision and CCD resolution determine the alignment error of the focal plane. Second, the align tilt or rotation errors with the laser interferometer, in which the tilt errors including the docking error are accumulated by the whole linear scale and *BD* error mentioned in Section 2.2, will be eliminated by calibrating the system projection dimension precision and adjusting the projection tilt error. Moreover, the rotation errors directly affect the pitch accuracy, which could be eliminated by the boundary expression and weight function of the alignment function. Moreover, it is also associated with the adjustment of the tilt error, and the same steps are repeated to reduce the error by multiple times, thereby achieving the desired results.

## 4. Alignment Experiments

### 4.1. Align the Focal Plane Based on the CCD Image

The purpose of the alignment experiment is to verify the accuracy of the CCD image alignment principle, ensure when the lithography is ready by precisely projecting the grating mask onto the photoresist plane, and form a higher resolution CCD image in the focal plane. It is needed to adjust the position of the CCD focal plane so that it coincides with the grating photoresist surface, then transfer the images in the focal plane to a computer, and assess the CCD image clarity with the evaluation function. The clearest picture is infinitely close to the focal plane of the projection motherboard and within the error range.

The characterization criteria of a picture clarity are: a single peak, steep, one-way. In this study, the application of the CCD image alignment technique is essentially the image gray value changes in the steepness. An *m* × *n* pixel image is determined by *X*[*m*], whereas *Y*[*n*] is composed of a combination of two-dimensional arrays. 

**Figure 8:**
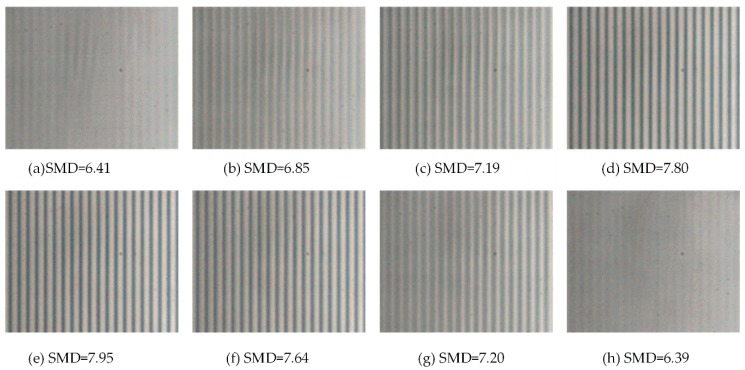
Detected images at different focal positions.

As shown in Figure 8a–f, the image acquisition of different focus positions is measured by a micro-CCD sensor. In the figures, the image is changing from fuzzy to clear, followed by an opposite clear to fuzzy change process. The gray value of the cross-section, $X\left[x_{0},m\right]$, is extracted by the gray value change curve extracted in Figure 8a–f, and then the adjacent pixel gray variance method is used and normalized. The maximum value is the clearest picture, and the focal length of the corresponding with its evaluation function are employed to obtain the value and normalized value listed in Table 1.

**Figure 9:**
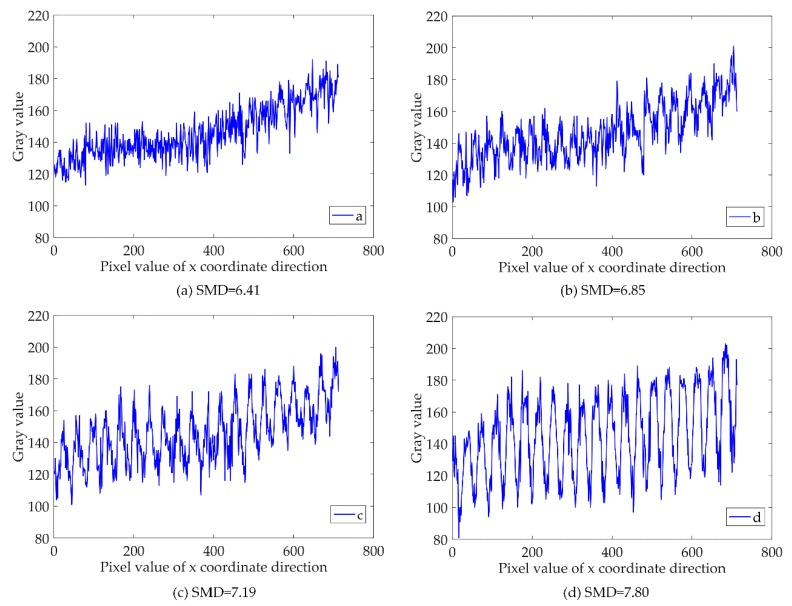

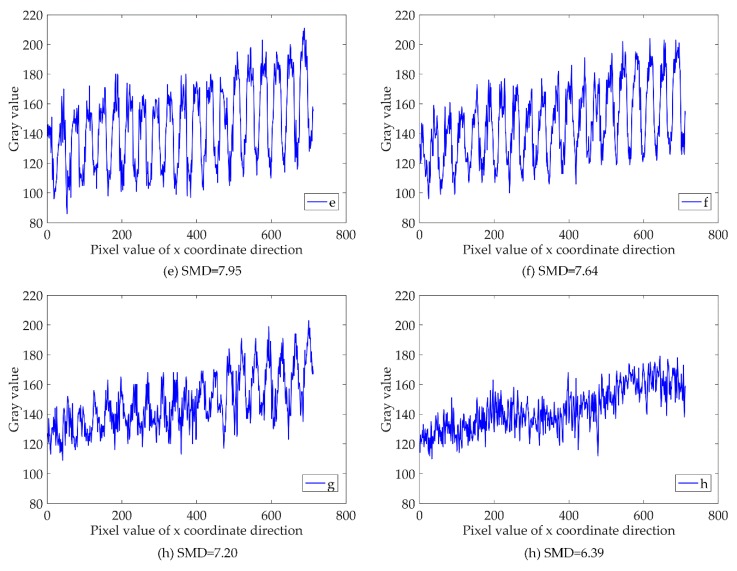
Gray value curves at different focal planes.

Figure 9 presents the extracted *X*[1, *m*] of the image curve of the gray value using MATLAB software. The clearest picture is identified by assessing the trend curve. The image shows that the grating strips are vertical and horizontal, showing the distribution of the lightness and darkness. The trend displayed by the gray values of each row in the image is the same, and for the evaluation resolution data selection, the first row of the array is simply chosen as a representative value.

**Figure 10:**
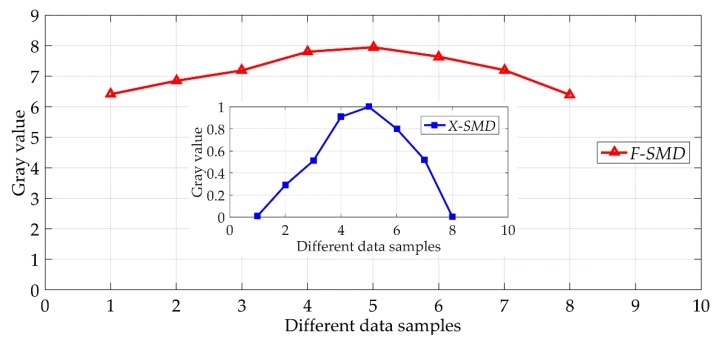
Gray value change trend.

From the above experimental results, especially as shown in Figure 10, it can be clearly seen that the criterion of image evaluation exhibits a strong single peak, clearest image can be distinguished, and alignment effect of the photolithography plane is realized, and accuracy of the grating lithography is improved objectively.

### 4.2. Align Tilt and Rotation Mask with Laser Interferometer

The principle of the mask plate tilt precision alignment is measuring the length using a dual-frequency laser interferometer and calibrating the lithography projection line width of the mask. Based on the precise alignment of the focal plane, the tilt mask is aligned using the laser interferometer, which is the linear displacement feedback device for Renishaw dual-frequency laser interferometer XL-80. The motor and drive use the Taiwan Delta AC servo motor. The laser beam of the dual-frequency laser interferometer is located on the same straight line as the projection surface of the mask, which accords with Abbe’s error principle. The CCD is used to collect the image, then an image processing technology in a computer is applied, and ultimately the photolithography process is used for the verification.

In the experiment, the common linear-scale projection lithography splice process is replaced by the average homogenization technique which overlaps the different position pitches regularly [13]. We use the splicing lithography method for alignment, and the lithography process includes coating, before drying, lithography, development, post-baking, hardening, and corrosion. The positive photoresist uses BP series, and the resolution is up to 0.1 μm.

**Figure 11:**
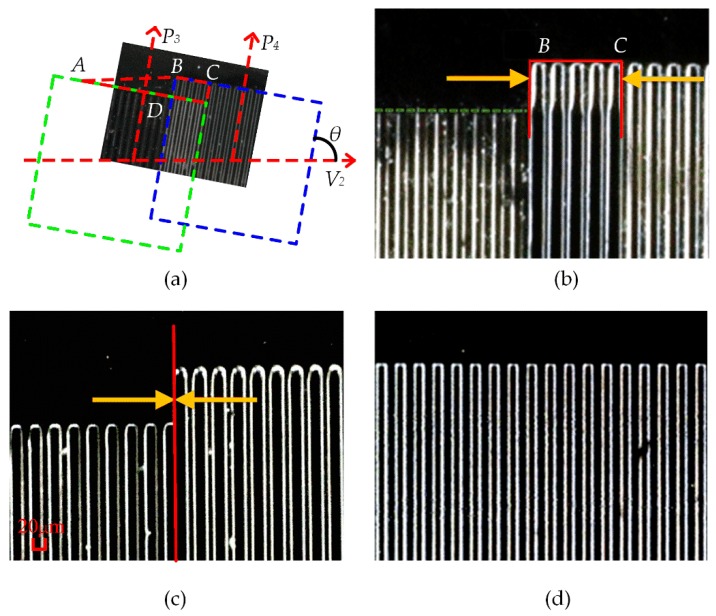
Several grating images of alignment: (**a**) Principle of the splicing lithography method (**b**) Overlaps of the first lithography (**c**) Overlaps of first lithography after adjusting (**d**) Third lithography after achieving precise alignment.

As shown in Figure 11a, $P_{3}$ is the first photolithographic position, moving through the control system and laser feedback system in the $V_{2}$ direction by 4.800 mm, reaching $P_{4}$ position and performing the photolithography, and applying the photolithography process for development. Three corrections, as included in Figure 11b,c, are performed based on the photolithography test to obtain the result shown in Figure 11d. By performing the focusing process in the above flow chart, the *BC* value is reduced to reach the error range and the alignment method is verified.

### 4.3. Influence of the Alignment Error on the Lithography Accuracy

**Figure 12:**
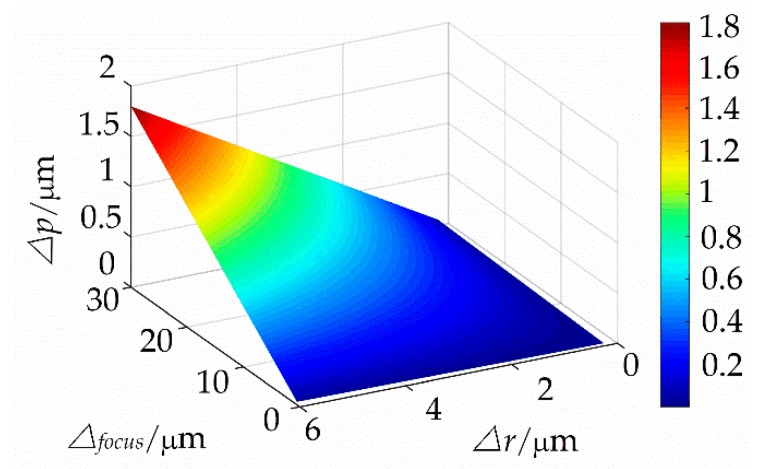
Relationship between the focal plane deviation and offset deviation of the lithography pitch.

The lithography precision alignment error discussed in this section is mainly for the static case, i.e., before the lithography process, a full-length static calibration of multiple points is conducted to achieve the focal length alignment. In contrast, in the grating lithography process, the main factors that affect the focal length alignment error include the movement straightness of the guideway direction, clamping error of the blank grating, and vibration of the moving process, which cause the deviation in the overlap of the focal plane and photoresist plane of the blank grating. For the static case, the relationship between the focal plane deviation and offset deviation of the lithography pitch is expressed in Equation (19) and depicted in Figure 12.

(1) When an alignment plane is away from the focal plane in the alignment process, it will result in the contrast problems, and it is easy to form deviation on the threshold plane.
(2) And when there is a rotation angle error, it will result in an inclination of the energy distribution.
  (19) $$\Delta p\propto \Delta_{focus},\Delta r$$
  where $\Delta_{focus}$ is the alignment error and Δr is the projection error due to angular rotation.

**Figure 13:**
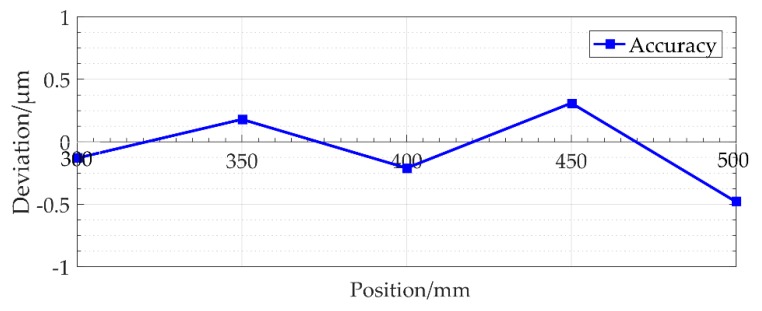
Grating lithography accuracy detection at 200-mm-length.

In the experimental verification, when the tilt angle is aligned as discussed previously in Section 4.2, consideration of the uncertain factors and laser displacement sensor pitch alignment yields a CCD maximum resolution pixel of 0.1 μm while reducing the effect of the other parameter factors. As shown in Figure 13, the accuracy of the scale is 0.79 μm in only a 200-mm-measurement range.

## 5. Conclusions

In this paper, we propose a method for improving the alignment accuracy of grating projection lithography. The principle and adjustment algorithms for the projection lithography are used to explain how to obtain the alignment accuracy. The simulation and experimental results are discussed, and the following conclusions can be drawn:

(1) The optimal position of the focal plane of the mask is determined by the CCD image, which is used to calculate the sharpness of the image using an image mathematical algorithm. The alignment algorithm is normalized to achieve precise alignment by adjusting the position of the lithography system.
(2) For plane alignment, four types of conventional tilt cases are listed, and a mathematical model is used to interpret the method. The plane tilt tolerance error is described from a theoretical perspective.
(3) For the rotation error alignment, the rectangular wave Fourier fundamental frequency algorithm of the lithography lens system is used to model the rotation errors, and the limit functions of the real and imaginary parts are described. Simultaneously, the frequency and phase are assigned the limit functions. Finally, the alignment accuracy function of the rotation error is characterized by the weighting of the errors.

The simulation analysis results confirm that the-dual frequency laser interferometer corrects the inclination of the motherboard so that it adjusts to the same direction as the table movement and achieves alignment accuracy of less than 0.1 μm. The experimental results verify the lithography alignment method, and the lithography process is performed based on a CCD and mathematical algorithm. The lithography accuracy of scale is 0.79 μm in only a 200-mm measurement range.

## Figures and Tables

**Table 1:** Adjacent Pixel Gray Variance and Normalization.

	a	b	c	d	e	f	g	h
*F-SMD*	6.41	6.85	7.19	7.80	7.95	7.64	7.20	6.39
*X-SMD*	0.01	0.29	0.51	0.91	1.00	0.80	0.52	0.00
